# Negative photoresponse in Ti_3_C_2_T_*x*_ MXene monolayers

**DOI:** 10.1515/nanoph-2022-0182

**Published:** 2022-07-14

**Authors:** Nataliia S. Vorobeva, Saman Bagheri, Angel Torres, Alexander Sinitskii

**Affiliations:** Department of Chemistry and Nebraska Center for Materials and Nanoscience, University of Nebraska – Lincoln, Lincoln 68588, NE, USA

**Keywords:** MXenes, negative photoresponse, Ti_3_C_2_T_*x*_, titanium carbide

## Abstract

Two-dimensional transition metal carbides, nitrides, and carbonitrides, collectively known as MXenes, are finding numerous applications in many different areas, including optoelectronics and photonics, but there is limited information about their intrinsic photoresponse. In this study, we investigated the visible and near-infrared range photoresponse of Ti_3_C_2_T_*x*_, the most popular MXene material to date. The electrical measurements were performed on devices based on individual monolayer Ti_3_C_2_T_*x*_ MXene flakes, which were characterized by a variety of microscopic and spectroscopic methods. For MXene devices with different electrode layouts, the current reproducibly decreased under illumination with either white light or lasers with different wavelengths in the visible and near-infrared region, thus demonstrating a negative photoresponse. The understanding of the intrinsic photoresponse of Ti_3_C_2_T_*x*_ should facilitate the optoelectronic and photonic applications of MXenes.

## 1 Introduction

MXenes are a rapidly growing family of two-dimensional (2D) transition metal carbides, nitrides, and carbonitrides that are finding numerous applications in many different areas ranging from energy storage and electromagnetic interference shielding to gas sensing and biomedicine [1]. There is also an active discussion of the potential use of MXenes for optoelectronic applications, such as photodetectors, phototransistors, and solar cells [2–7]. In many of the reported optoelectronic devices, MXenes were used in combination with various semiconductor materials, such as TiO_2_ [8], CdS [9], MoS_2_ [10], CH_3_NH_3_PbI_3_ perovskite [11], ZnO [12], and others. Several of these devices demonstrated enhanced characteristics or intriguing physical properties, such as a negative photoresponse in photodetectors based on combinations of Ti_3_C_2_T_*x*_ MXene with CdS [9] and MoS_2_ [10]. However, when analyzing the behavior of heterostructural or composite materials it is imperative to fully understand the intrinsic properties of their individual components, and while the optoelectronic properties of the above semiconductors have been extensively studied, there is limited information on the intrinsic photoresponse of MXenes.

In this study, we investigated the visible and near-infrared range photoresponse of Ti_3_C_2_T_*x*_ (T_*x*_ corresponds to the surface termination of the 2D Ti_3_C_2_ sheets), the most popular MXene material to date [13]. We performed the measurements on individual Ti_3_C_2_T_*x*_ flakes to exclude the interfacial phenomena at the contacts between the MXene flakes in their bulk assemblies, such as thin films. The measurements showed that devices based on individual monolayer Ti_3_C_2_T_*x*_ MXene flakes exhibit a negative photoresponse, *i.e*. the current decreased when the devices were illuminated with either white light or a laser in the visible or near-infrared range of spectrum. This effect is very different from the behavior previously observed for the partially oxidized Ti_3_C_2_T_*x*_ MXene, in which the presence of TiO_2_, a wide bandgap semiconductor, resulted in a positive photoresponse to ultraviolet (UV) light, so that the device current increased under illumination [8]. Consequently, the understanding of the intrinsic photoresponse of Ti_3_C_2_T_*x*_ will be beneficial for designing new MXene composites with other semiconductor materials for a variety of optoelectronic applications [2–4].

## 2 Experimental section

### 2.1 Synthesis of Ti_3_C_2_T_*x*_ MXene

Ti (99%, 325 mesh), Al (99%, 325 mesh), and TiC (99.9%, 325 mesh) were purchased from Alfa Aesar. HCl was purchased from VWR, and LiF was purchased from Spectrum Chemical. All chemicals were used as received. Ti_3_AlC_2_ MAX phase was synthesized using the 2:1:1.2 M ratio of TiC:Ti:Al. All precursors were mixed using a pestle and mortar, pressed into a pellet at 3500 psi, and annealed at 1450 °C for 8 h under the flow of argon (300 sccm). After the synthesis, Ti_3_AlC_2_ MAX phase was crushed and sieved to collect uniformly sized particles.

Ti_3_C_2_T_*x*_ MXene was synthesized by the minimally intensive layer delamination (MILD) method [14]. In brief, the MAX phase (500 mg, particle size below 38 µm) was slowly dispersed in the mixture of LiF (800 mg) and 10 mL of HCl (9 M), and stirred for 24 h (600 rpm, 25 °C). The sample was washed and centrifuged until pH 6 was reached and then delaminated into flakes by shaking for 15 min. Finally, the shaken solution was centrifuged (3500 rpm, 30 min), and single-layer flakes in the supernatant were stored at 4 °C for further experiments.

### 2.2 Materials characterization

Scanning electron microscopy (SEM) was performed using a FEI Nova NanoSEM instrument at the accelerating voltage of 5 kV. Raman spectra of Ti_3_C_2_T_*x*_ MXene were recorded using a Thermo Scientific DXR Raman microscope with a 532 nm excitation laser. Transmission electron microscopy (TEM) and selected area electron diffraction (SAED) of Ti_3_C_2_T_*x*_ flakes were performed using a FEI Tecnai Osiris scanning transmission electron microscope equipped with a HAADF detector and an X-FEG high brightness Schottky field-emission gun; the accelerating voltage was 200 kV. Powder X-ray diffraction (XRD) patterns were collected using a Rigaku Smart Lab powder diffractometer with Ni-filtered Cu Kα radiation operated at 40 kV and 30 mA, using 0.03° step and 3 s dwelling time. Ultraviolet–visible–near infrared (UV−vis−NIR) absorption spectrum of an aqueous solution of Ti_3_C_2_T_*x*_ MXene flakes was recorded using a Jasco V-670 spectrophotometer. Atomic force microscopy (AFM) of Ti_3_C_2_T_*x*_ MXene devices was performed with a Bruker Dimension Icon atomic force microscope using PeakForce Tapping mode.

### 2.3 Device fabrication and electrical measurements

A droplet of an aqueous solution of Ti_3_C_2_T_*x*_ MXene flakes was placed on a surface of a p-doped Si substrate covered with a 300-nm-thick layer of SiO_2_. The droplet was dried in air, resulting in Ti_3_C_2_T_*x*_ MXene flakes dispersed over the Si/SiO_2_ substrate. A Zeiss Supra 40 field-emission scanning electron microscope and a Raith pattern generator were used for electron beam lithography (EBL) to pattern electrodes on the Ti_3_C_2_T_*x*_ MXene flakes. An AJA electron beam evaporator at the base pressure of ∼8 × 10^−9^ Torr was used to evaporate 3 nm of Cr and then 20 nm of Au, both at the 0.02 nm/s rate.

The Ti_3_C_2_T_*x*_ MXene devices were measured in a Lake Shore TTPX cryogenic probe station at the base pressure of about 2 × 10^−6^ Torr. Prior to the electrical measurements, the devices were kept in the evacuated chamber of a probe station for at least 2 days to minimize the effect of surface adsorbates [15]. The electrical measurements were performed using an Agilent 4155C semiconductor parameter analyzer that was operated using a National Instruments LabView code.

For the white light illumination of the Ti_3_C_2_T_*x*_ MXene devices, we used a 150 W Philips 14,501 DDL 20 V halogen light bulb; the emission spectrum of this bulb was reported elsewhere [16]. A Thorlabs S120C standard photodiode power sensor was used to measure the maximum light intensity at 600 nm of 6 mW cm^−2^.

For the wavelength-dependent photoconductivity measurements, we used a Thorlabs multichannel laser source with 517 nm (green), 686 nm (red), and 965 nm (infrared) outputs. For each laser, the power was standardized at 3.5 mW. A laser spot with a diameter of about 5 mm was aligned on a Ti_3_C_2_T_*x*_ MXene device using an optical fiber.

## 3 Results and discussion

**Figure 1:**
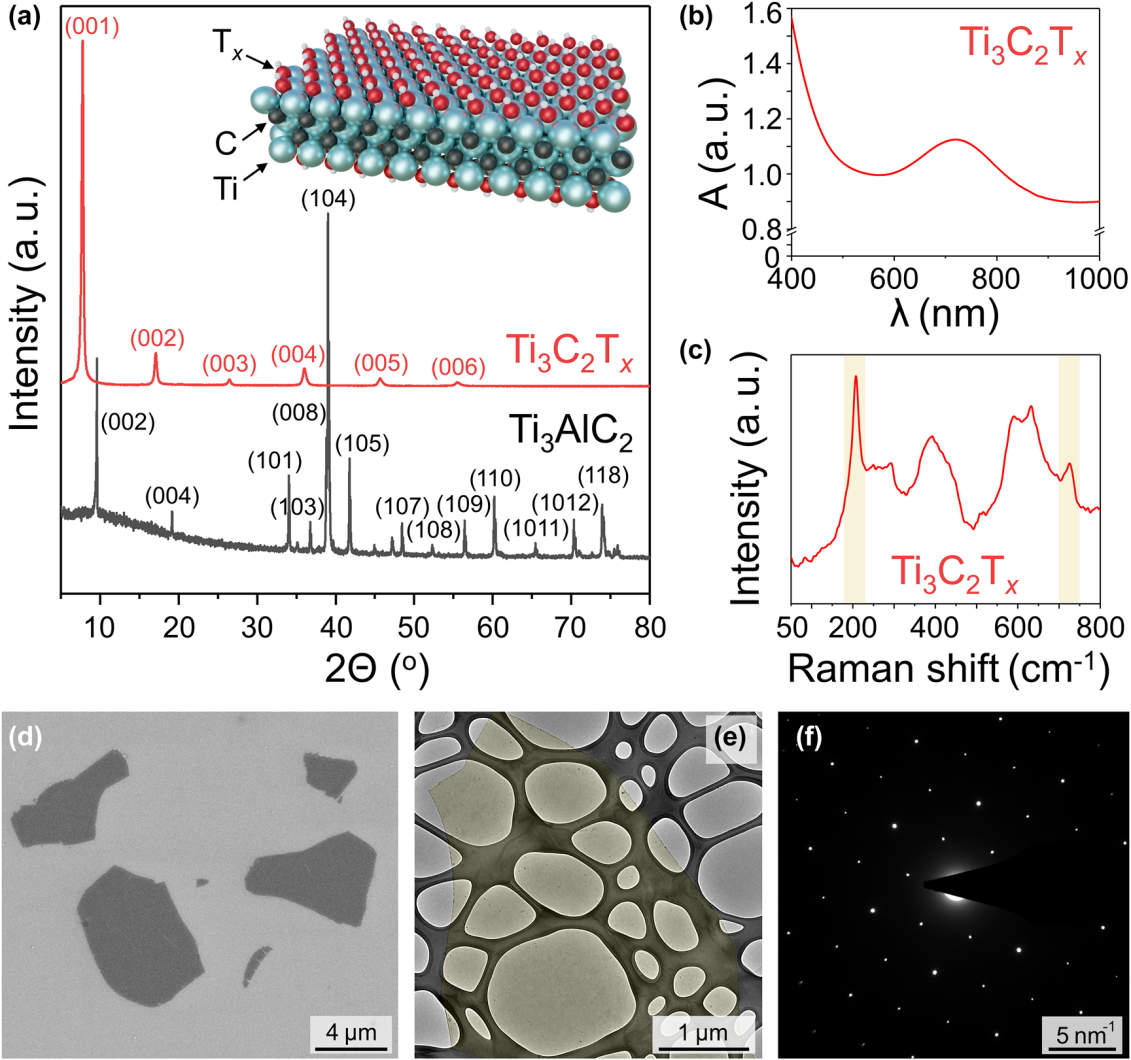
Characterization of Ti_3_C_2_T_*x*_ flakes. (a) XRD patterns collected for the Ti_3_AlC_2_ MAX phase powder (black) and the Ti_3_C_2_T_*x*_ MXene film (red). The inset shows the structure of a Ti_3_C_2_T_*x*_ MXene monolayer; Ti – blue spheres, C – black spheres, the surface functional groups (T_*x*_) are shown as –OH groups. (b) UV–vis-NIR absorption spectrum of an aqueous solution of Ti_3_C_2_T_*x*_ MXene flakes. (c) Raman spectrum of Ti_3_C_2_T_*x*_ MXene flakes on a gold-covered silicon substrate. (d) SEM image of Ti_3_C_2_T_*x*_ MXene flakes on a silicon substrate. (e) TEM image of a monolayer Ti_3_C_2_T_*x*_ flake on a lacey carbon grid. The flake is colored in yellow for clarity. (f) SAED pattern of the monolayer Ti_3_C_2_T_*x*_ flake shown in panel (e).

The inset in Figure 1a shows the structure of a monolayer Ti_3_C_2_T_*x*_ MXene flake. The flake contains three hexagonal layers of titanium atoms arranged in the cubic close-packed arrangement, in which the carbon atoms occupy the octahedral voids. The Ti_3_C_2_T_*x*_ flakes are known to be terminated by a variety of functional groups, such as –OH, =O, and –F [17, 18], which enable their solubility in water.

The formation of Ti_3_C_2_T_*x*_ MXene from Ti_3_AlC_2_ MAX phase is illustrated by the XRD patterns in Figure 1a. The black XRD pattern corresponds to Ti_3_AlC_2_ MAX phase and is consistent with the literature data [14]. No Ti_3_AlC_2_ peaks are observed in the XRD pattern of the prepared Ti_3_C_2_T_*x*_, suggesting the absence of appreciable quantities of MAX phase residues in the synthesized MXene. The only diffraction peaks observed in the red XRD pattern are the 00*l* (*l* = 1, 2, … 6) reflections, indicating a layered structure of stacked Ti_3_C_2_T_*x*_ MXene flakes with an interplanar distance of about 1.25 nm. This value is larger than the nominal thickness of a Ti_3_C_2_T_*x*_ monolayer of 0.98 nm [19]. The difference in these values suggests the presence of the water molecules trapped between the layers in bulk MXene assemblies, which is consistent with the previously reported data on Ti_3_C_2_T_*x*_ synthesized using the MILD method [14].

Figure 1b shows a UV–vis-NIR absorption spectrum of an aqueous solution of Ti_3_C_2_T_*x*_ MXene flakes. The spectrum demonstrates that Ti_3_C_2_T_*x*_ MXene has strong optical absorption in the visible and NIR range of spectrum. There is an absorption minimum at about 520 nm in the visible range, which is consistent with the dark green color of an aqueous solution of Ti_3_C_2_T_*x*_ MXene [20, 21]. The absorption maximum between 700 and 800 nm was reported to represent a plasmon resonance in Ti_3_C_2_T_*x*_ MXene [22, 23].

Figure 1c shows a Raman spectrum of Ti_3_C_2_T_*x*_ MXene flakes deposited on a gold-covered Si/SiO_2_ substrate; the measurement was performed using a 532 nm excitation laser. The spectrum shows two characteristic sharp peaks at about 208 and 726 cm^−1^, which are highlighted in Figure 1c, that correspond to the out-of-plane A_1g_ modes of a Ti, C and T_*x*_ group vibration and a C vibration, respectively [24, 25]. The Raman spectra of MXenes were reported to strongly depend on the excitation laser energy [26]. In particular, a use of a laser with an energy coupled with the plasmon resonance peak of Ti_3_C_2_T_*x*_ would enhance the 726 cm^−1^ peak and also reveal the resonant Raman peak around 120 cm^−1^, which was described as an in-plane E_g_ group vibration of Ti, C, and T_*x*_ [24–26]. The 230 to 470 cm^−1^ range was described as a T_*x*_ region [25] containing in-plane E_g_ vibrations that depend on the identity of the surface functionalities. The peaks in the region from 580 to 730 cm^−1^ are primarily carbon in-plane and out-of-plane vibrations [25]. Raman spectra of Ti_3_C_2_T_*x*_ samples strongly depend on the synthetic method and the environment of the MXene flakes [24–26]. The observed spectrum does not correspond to the Raman spectrum of Ti_3_AlC_2_ [27], further confirming the transformation of the precursor MAX phase to Ti_3_C_2_T_*x*_ MXene.

The Ti_3_C_2_T_*x*_ MXene flakes were visualized by SEM and TEM. Figure 1d shows SEM image of several Ti_3_C_2_T_*x*_ MXene flakes that were deposited on a Si/SiO_2_ substrate from an aqueous solution. The flakes look uniform and, with their lateral dimensions in a few µm range, are sufficiently large for device fabrication by EBL. Another Ti_3_C_2_T_*x*_ MXene flake is shown in TEM image in Figure 1e. The flake was imaged on a lacey carbon TEM grid, which is seen as a dark weblike structure, and it was colored in yellow for better visibility. In the case of partial oxidation of Ti_3_C_2_T_*x*_ flakes, microscopy analysis often reveals pinholes and elongated TiO_2_ particles, which are typically observed along the flake edges [28]. In our case, the imaged flake has a uniform surface and does not display pinholes and foreign particles, suggesting the high quality of the MXene material. The high quality of the MXene flake is further confirmed by the hexagonal SAED pattern (Figure 1f), which is consistent with the structure of Ti_3_C_2_T_*x*_ shown in the inset in Figure 1a.

**Figure 2:**
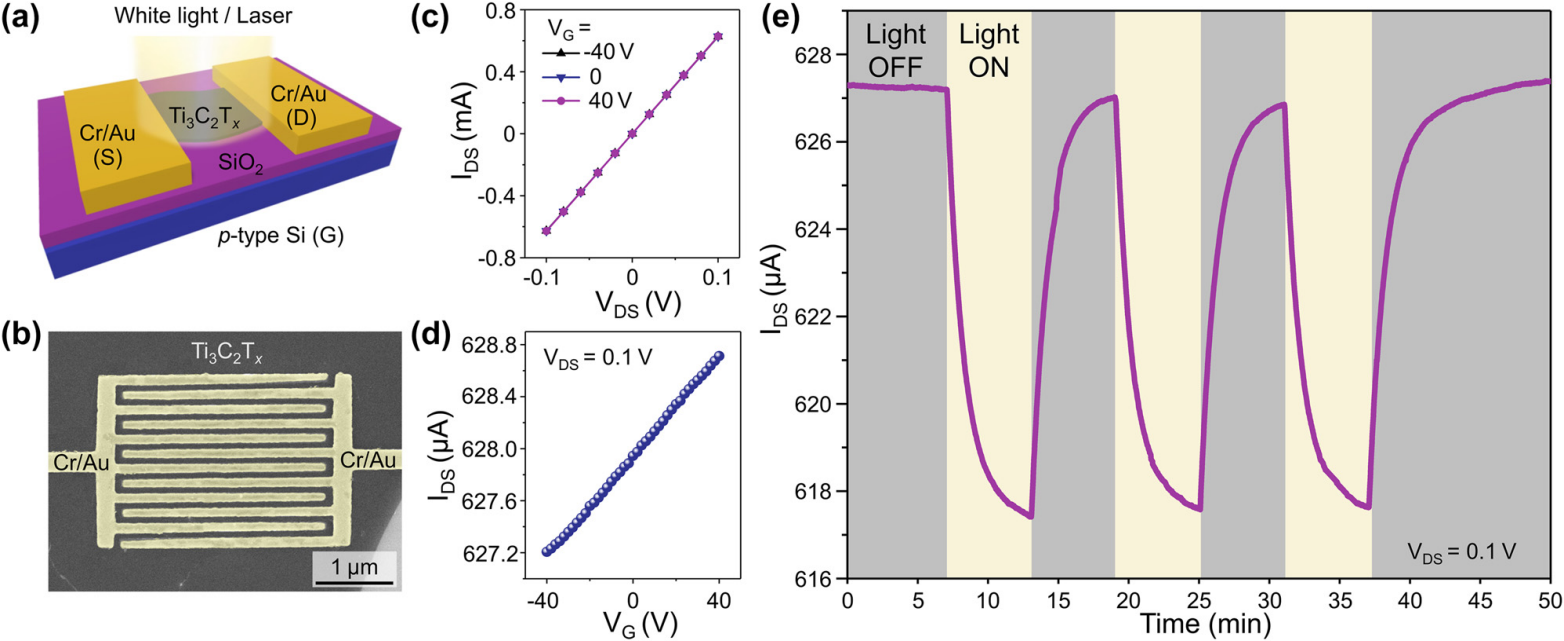
Negative photoresponse of monolayer Ti_3_C_2_T_*x*_ flakes to white light. (a) Scheme of a two-terminal Ti_3_C_2_T_*x*_ device under illumination. (b) SEM image of a device with interdigitated electrodes patterned on a monolayer Ti_3_C_2_T_*x*_ MXene flake. The electrodes are colored in yellow for clarity. (c) *I*_DS_–*V*_DS_ curves obtained from two-terminal measurements of a Ti_3_C_2_T_*x*_ device shown in (b) at the gate voltages of −40, 0, and 40 V. (d) *I*_DS_–*V*_G_ dependence for the same device measured at *V*_DS_ = 0.1 V. (e) Negative photoresponse of the same device to white light, measured at *V*_DS_ = 0.1 V and *V*_G_ = 0 V.

The general scheme of the Ti_3_C_2_T_*x*_ devices tested in this study is shown in Figure 2a. The scheme shows a monolayer Ti_3_C_2_T_*x*_ flake bridging source (S) and drain (D) Cr/Au electrodes on a Si/SiO_2_ substrate. The heavily p-doped Si substrate served as a global bottom gate (G) electrode in the electrical measurements. For the illumination of the devices, we used either white light of a halogen bulb or one of the lasers. We fabricated numerous devices using Ti_3_C_2_T_*x*_ MXene flakes from three different batches and employed several different device geometries, and a negative photoresponse was observed in all experiments.

One of the tested device structures is shown in SEM image in Figure 2b, where we used interdigitated Cr/Au electrodes fabricated on a large monolayer Ti_3_C_2_T_*x*_ MXene flake. Figure 2c shows the drain-source current (*I*_DS_) – drain-source voltage (*V*_DS_) dependences measured for this device at the gate voltage (*V*_G_) of −40, 0, and 40 V. The *I*_DS_–*V*_DS_ dependences are linear, which is indicative of Ohmic contacts between the MXene flake and the Cr/Au electrodes. The *I*_DS_–*V*_DS_ dependences measured at different gate voltages nearly overlap, indicating a weak modulation of the electrical conductivity of Ti_3_C_2_T_*x*_ by the gate voltage. Figure 2d shows transfer characteristics measured in the range of −40 V to +40 V applied to the gate electrode. The *I*_DS_ gradually increases when *V*_G_ sweeps from negative to positive values, indicating the n-type behavior. These *I*_DS_–*V*_DS_ and *I*_DS_–*V*_G_ dependences are consistent with the results of electrical measurements reported for monolayer Ti_3_C_2_T_*x*_ MXene devices in our previous works [14, 29].

Figure 2e shows the results of photoconductivity measurements of the same monolayer Ti_3_C_2_T_*x*_ device. We used a vacuum probe station chamber with an optical window that could be either closed or opened to enable the illumination of a device. When the window was closed and the device was kept in the dark, it showed a drain-source current of about 627 μA at *V*_DS_ = 0.1 V and *V*_G_ = 0 V. However, when the window was opened, enabling the illumination of the device with a white light of a halogen bulb, the drain-source current decreased by about 10 µA, demonstrating that monolayer Ti_3_C_2_T_*x*_ MXene exhibits a negative photoresponse. This effect was completely reversible, and once the window was closed, the drain-source current increased back to the original value of about 627 µA. The optical modulation of *I*_DS_ in MXene devices is reproducible, which is demonstrated by three consecutive switching cycles in Figure 2e that look nearly identical.

**Figure 3:**
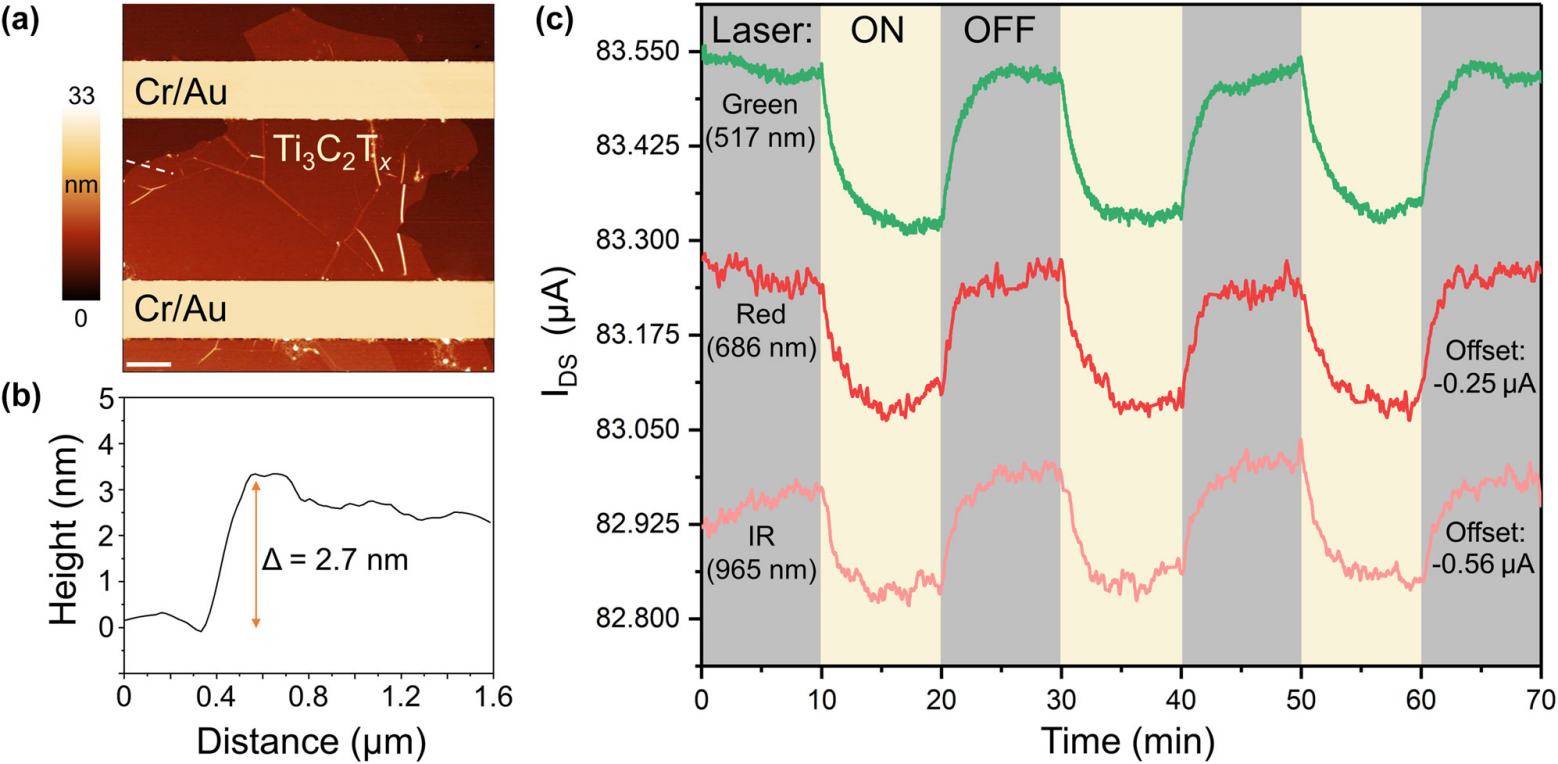
Negative photoresponse of monolayer Ti_3_C_2_T_*x*_ flakes to lasers with different wavelengths. (a) False-color AFM image of a two-terminal device based on a monolayer Ti_3_C_2_T_*x*_ MXene flake. Scale bar: 1 μm. The Cr/Au electrodes are colored in yellow for clarity. (b) AFM height profile measured along the white dashed line in panel (a). (c) The modulation of photocurrent in Ti_3_C_2_T_*x*_ device with three different lasers, IR (965 nm), red (686 nm), and green (517 nm). All lasers were set to the same power of 3.5 mW. The measurements were performed at *V*_DS_ = 0.1 V and *V*_G_ = 0. The *I*_DS_ axis corresponds to the electrical measurement involving the green light; the other two dependences are vertically offset for clarity by −0.25 μA (red laser) and −0.56 μA (IR laser), respectively.

Figure 3a shows a different device that employed a monolayer Ti_3_C_2_T_*x*_ MXene flake from a different batch and a simpler two-terminal device geometry that is identical to the structure schematically shown in Figure 2a. This device was visualized by AFM to confirm the monolayer thickness of the MXene flake. The AFM height profile measured along the white dashed line in Figure 3a shows a step height of about 2.7 nm, see Figure 3b. It should be noted that according to theoretical calculations and high-resolution TEM studies, the nominal thickness of a Ti_3_C_2_T_*x*_ MXene monolayer is 0.98 nm [30, 31]. However, previous AFM height measurements of monolayer Ti_3_C_2_T_*x*_ MXene flakes on Si/SiO_2_ substrates also yielded thicknesses of about 2.7 nm [14, 19, 28], and the increased height was explained by the presence of surface adsorbates, such as water molecules, that are trapped under the flakes. Similarly, increased thicknesses of monolayer flakes in AFM measurements were also reported for other MXenes [32, 33] and various 2D materials [34–37].

Figure 3c shows that negative photoresponse was observed not only when the monolayer Ti_3_C_2_T_*x*_ MXene devices were illuminated with white light, but also in experiments involving different lasers with wavelengths in the visible and near-infrared range of spectrum. We used lasers with wavelengths of 517, 686 and 965 nm, at all of which Ti_3_C_2_T_*x*_ MXene exhibits a considerable optical absorption. Figure 3c shows that the drain-source current reproducibly decreased when the device was illuminated with any of the three lasers and then restored back to the original value once the optical window was closed. The negative photoresponse observed in these experiments can be explained by the metallic nature of the electrical conductivity of Ti_3_C_2_T_*x*_ MXene [38, 39], which decreases under the illumination due to increased scattering. While we did not have access to a laser with a wavelength in the 700–800 nm range that would match the plasmon resonance in Ti_3_C_2_T_*x*_ MXene [22, 23], it would be interesting to investigate whether the use of such laser would result in an enhanced negative photoresponse due to an increased absorption.

The negative photoresponse makes metallic Ti_3_C_2_T_*x*_ MXene very different from a great variety of semiconducting 2D materials that had been extensively studied for photodetector applications in recent years and exhibited positive photoresponse [40–46]. The negative photoresponse in pristine Ti_3_C_2_T_*x*_ is also very different from the positive photoresponse of the partially oxidized material, in which the oxidation of Ti_3_C_2_T_*x*_ results in the formation of TiO_2_, a wide bandgap semiconductor that could be excited with UV light [8]. Collectively, these results suggest that Ti_3_C_2_T_*x*_ could exhibit a tunable photoresponse that would depend on the degree of oxidation of MXene.

## 4 Conclusions

In summary, we demonstrated that individual monolayer Ti_3_C_2_T_*x*_ MXene flakes exhibit a negative photoresponse in the visible and near-infrared spectrum range. This information is important for several areas of MXene research. First, there are ongoing studies of MXenes that aim to determine their intrinsic electrical properties, such as electrical conductivity [14, 21, 29, 32] and breakdown current density [29, 33]. The knowledge of these properties is necessary for advancing a variety of MXene applications ranging from energy storage [47] and transparent conductive electrodes [48, 49] to interconnects [29, 33] and gas sensors [28, 39]. Since this study establishes that the conductivity of Ti_3_C_2_T_*x*_ (and likely, other MXenes) is affected by light, it should be important for the future electrical property characterizations of MXenes to indicate whether the measurements were performed under illumination or in the dark for a more accurate comparison of results from different studies. Second, the negative photoresponse of Ti_3_C_2_T_*x*_ MXene could be responsible for a similar effect reported for MXene heterostructures with other materials [9, 10]. Finally, the negative photoresponse of Ti_3_C_2_T_*x*_ should be taken into account in MXene applications that directly employ light–matter interactions, such as photodetectors and solar cells [2], [3], [4, 11].
